# Anticancer effects of herbal medicine Emelia-M, Mshikazi and Delosma H against human leukaemia cells

**DOI:** 10.4314/ahs.v23i2.35

**Published:** 2023-06

**Authors:** Joy Nkechinyere Adeniyi, Manimbulu Nlooto, Mlungisi Ngcobo, Roshila Moodley, Exnevia Gomo

**Affiliations:** 1 Traditional Medicine Laboratory, School of Nursing and Public Health, College of Health Sciences, University of KwaZulu-Natal; 2 Department of Pharmacy, School of Health care Sciences, University of Limpopo; 3 Department of Chemistry, School of Physics and Chemistry, University of KwaZulu-Natal

**Keywords:** Leukaemia, traditional herbal medicines, apoptosis, phytochemical compounds

## Abstract

**Background:**

Leukaemia is one of the three major types of blood cancers that lead to the overproduction of abnormal white blood cells. *Emelia M* (EMB), *Mshikazi* and *Delosma H* are herbal medicines that are being used by traditional healers in KwaZulu-Natal, South Africa to treat leukaemia and other diseases.

**Objectives:**

To gain insight into the safety (non-toxic effect), anti-cancer activity, mechanisms of action and phytochemical profiles of traditional herbal medicines *(Emelia M (EMB), Mshikazi* and *Delosma H)* in South Africa.

**Methods:**

The viability of human peripheral blood mononuclear cells (PBMCs), monocytic (THP-1) and T-lymphocyte (Jurkat) cell lines exposed to varying concentrations of aqueous extracts of the three herbal medicines were assessed using adenosine triphosphate (ATP) assay.

**Results:**

All three extracts showed a dose-dependent effect on the viability of PBMCs. Cell viability decreased with increasing concentrations of extracts when compared with the untreated cells at 24 and 48 hours. The inhibitory activities (IC50) of the extract were found in the order of *Mshikazi > EMB, > Delosma H*. All the extracts induced apoptosis with minimal necrosis. Many bioactive compounds that have been previously reported to have anticancer effects were identified in the extracts.

**Conclusion:**

*Mshikazi* extract significantly inhibited the growth of THP-1 and Jurkat cells and induced cell death through apoptosis than the other two extracts.

## Background

Cases of cancer continue to increase in Africa due to population growth and the prevalence of risk factors that can be linked to the economic transition 1,2. Leukaemia is blood-related cancer that affects the bone marrow, blood cells and parts of the lymphatic system. The two main forms of leukaemia are acute leukaemia which is known to advance rapidly with many immature white blood cells, and chronic leukaemia which advances more slowly and has more mature white blood cells 3. These can be further classified into two types which are myeloid and lymphoblastic leukaemia 4.

Although the cause of leukaemia is unclear, several risk factors have been identified including environmental factors 5, smoking, obesity, physical inactivity 1, exposure to benzene and certain chemotherapies, inherited syndromes and some viral infections 6. Significant progress has been made in the treatment and prevention of leukaemia 7 which includes chemotherapy, radiation therapy, biological therapy, and stem cell transplants 6,8. Despite this progress, treatment still has limitations including severe side effects 9.

Medicinal plants have been used to prevent and treat diverse diseases including cancer 10–12. Some medicinal plants which have been shown to have anticancer properties include *Amorphophallus campanulatus, Artemisia vulgaris, Bergenia ciliate* 13, *Boswellia serrata, Allium sativum* 14, *Moringa oleifera* 15 and *Catharanthus roseus* 16. Medicinal plants are the main sources of bioactive compounds which have significantly contributed to the discovery of various conventional drugs 12. Vinblastine and vincristine are potent anticancer drugs from *Catharanthus roseus* 17–19 and Taxol is an effective anticancer drug from the plant *Taxus brevifolia* 12,20,21. The use of natural plants has increased rapidly due to their perceived minimal side effects, safety and efficacy 9.

This study evaluated the anti-leukaemia activity and possible mechanisms of action of the extracts of *Delosma H, Mshikazi and Emelia* M (EMB) traditional herbal medicines on THP-1 monocyte and Jurkat lymphocyte leukaemia cell lines which have been widely used to screen anti-cancer plants 22–24. The study also evaluated the effect of the extracts on the viability of normal human peripheral blood monocytes (PBMC) and the phytochemical profiles of the extracts.

## Materials and Methods

### Study design

This was an experimental laboratory-based study that aimed to evaluate the extracts from three traditional herbal medicines, for their safety, efficacy, mechanism of action on leukaemia cancer cell lines, and also their phytochemical analysis using liquid chromatography-mass spectrophotometry.

### Plant materials and preparation

The three traditional herbal medicines (THMs) were provided by three traditional healers in Durban, South Africa, in 2017. The trade names of the three THMs are *Emelia-M* (EMB), *Mshikazi* and *Delosma H* which were made from a mixture of different medicinal plants. The names of the plants have been withheld to protect the intellectual property of the knowledge holders. However, the specific parts of the plants and areal parts were appropriately preserved for botanical verification. The THMs were provided as ready-to-use aqueous extracts.

The extracts were filter-sterilised using an Automatic Lid Clock (SP Scientific, USA) centrifuge at a maximum of 3 700 rpm for 10 minutes. Thereafter, each of the three extracts was freeze-dried to powder using a freeze dryer (SP Scientific, USA). All the extracts were weighed and kept in the freezer (-20^o^C) for long-term storage. A stock solution of each herbal extract was prepared using 100 mg of the powdered material dissolved in 10 mL of phosphate-buffered saline (PBS) to make a stock solution of 10 mg/mL. Working concentrations of each extract were prepared from the stock solution for cell viability assays.

### Cell lines

THP-1 cells are human monocytic leukaemia cells derived from the peripheral blood of a male with acute monocytic leukaemia and Jurkat cells are acute T cell human lymphoblastic leukaemia cells. The THP-1 cell lines were obtained from the Medical Microbiology Laboratory, University of KwaZulu-Natal (UKZN, Durban) and Jurkat T lymphocyte cells were donated by Dr Bongiwe Ndlovu from the UKZN HIV Pathogenesis Programme. Peripheral blood mononuclear cells (PBMCs) were provided by Dr Jacobus Hendricks, Human Physiology, School of Laboratory Medicine and Medical Sciences, UKZN.

### Preparation of assays

The assays that were prepared in this study are the Tissue culture assay; PBMC viability assay, THP-1 and Jurkat cell viability assays, ATP assays, Caspase 3/7 assay, Caspase 8 assay and DNA fragmentation assay. We also carried out Flow cytometry. The details about all of these methods are in the supplementary material.

### Liquid chromatography-mass spectrophotometry (LC-MS)

EMB, *Mshikazi*, and *Delosma H* were analysed on an ABSCIEX 4000 QTRAP hybrid triple quadrupole mass spectrometer with Shimadzu's front end. Twenty microliters of each sample were injected onto a Discovery C18 reverse-phase column (150 × 2.1 mm, Supelco) and separated using 0% to 95% linear water (Solvent A) and methanol (Solvent B) gradient over 30 minutes at 0.3 mL/min. Eluting analytes were first analysed in positive and then negative ionization mode, each time, using an information-dependent acquisition (IDA) method where ions between 200 and 1000 Da with intensities above 100 000 counts per second (cps) originating from an enhanced MS (EMS) survey scan were selected and fragmented in the collision cell and the fragments recorded following and enhanced production (EPI) scan. The LC-MS raw spectra were analysed and the matching molecules were predicted using automated mass spectral deconvolution & identification system (AMDIS) version 2.73 package obtained from NIST (25).

### Statistical analysis

Data analyses were done on Microsoft Excel (Microsoft Corporation, City, USA) to obtain descriptive statistics and IC50 values. The differences between groups of extracts were analysed using one-way analysis of variance (ANOVA) and the differences between the treated cells and the control cells were analysed using GraphPad Prism software (version 5) with the Tukey-Kramer multiple comparison post-hoc test. Differences with P ≤ 0.05 were considered statistically significant.

## Results

### In vitro cytotoxicity of the THMs extracts on normal PBMCs cells

**Figure 1:**
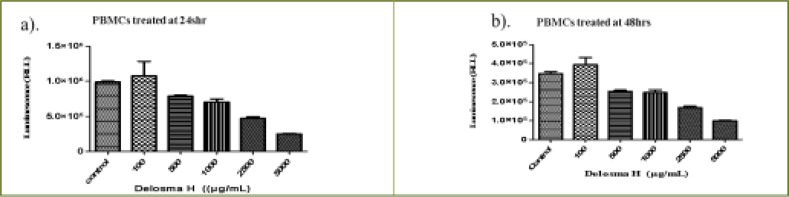
Effect of varying concentrations of *Delosma H* extract on the viability of human peripheral blood mononuclear cells (PBMCs) at (a) 24 h (b) 48 h

The cells were treated with different concentrations of the three extracts for 24 and 48 h. The viability of PBMCs was determined using the ATP assay. *Delomas H* extracts (100 µg/mL to 5.0 mg/mL) significantly decreased cell viability as the concentrations increased at 24h (P < 0.0003) and 48h (P < 0.0001) (Figure 1). The (IC50) values obtained at 24 and 48 h were 2268.7 µg/mL and 2165.6 µg/ml, respectively (Table 1).

**Table 1:** Half maximal inhibitory concentrations (IC50) of the three herbal extracts on PBMCs over 24 and 48 h

Traditional Herbal Medicine	(IC50) at 24 h (µg/mL)	(IC50) at 48 h (µg/mL)
*Delosma H (muthi 1)*	2268.7	2165.6
*Mshikazi (muthi 2)*	134.3	67.5
EMB *(muthi 3)*	1954.0	1401.6

**Figure S1:**
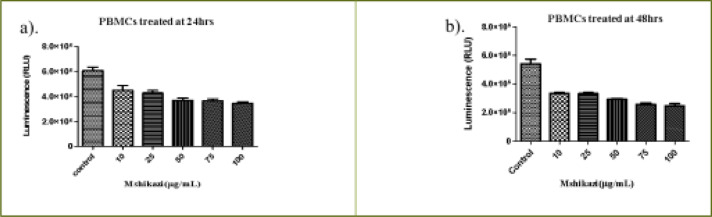
Effect of varying concentrations of Mshikazi extract on the viability of human peripheral blood mononuclear cells (PBMCs) at (a) 24 hours (b) 48 hours

*Mshikazi* extract at the concentrations range of 10 to 100 µg/mL showed a similar and significant decrease in the cell viability with increasing concentrations of extract (P < 0.0001) at both 24 and 48 h (Figure S1). The (IC50) values at 24 and 48 hours were 134.3 µg/mL and 67.5 µg/mL, respectively (Table 1).

**Figure S2:**
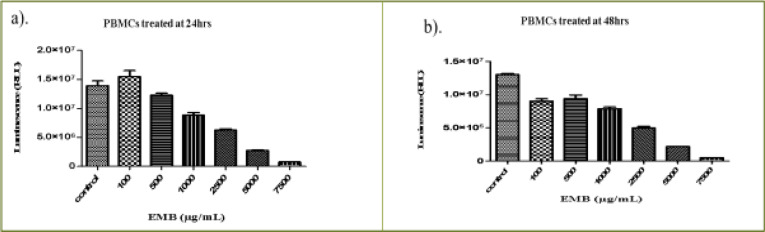
The effect of EMB extract on the viability of human peripheral blood mononuclear cells (PBMCs) (a) 24 hours (b) 48 hours

The EMB extract at concentrations ranging from 100 µg/mL to 7.5 mg/mL also significantly decreased the cell viability with increasing concentrations of extract (P < 0.0001) (Figure S2). The (IC50) values based on the range of the concentrations were 1954.0 µg/mL and1401.6 µg/mL, respectively for 24 and 48 hours as shown in Table 1.

### Effect of Extracts on Viability of THP-1 and Jurkat Cells

The (IC50) concentrations of the extracts were used to assess the viability of THP-1 and Jurkat cancer cell lines after 6, 24 and 48 hours of exposure.

### Delosma H extract

**Figure 2:**
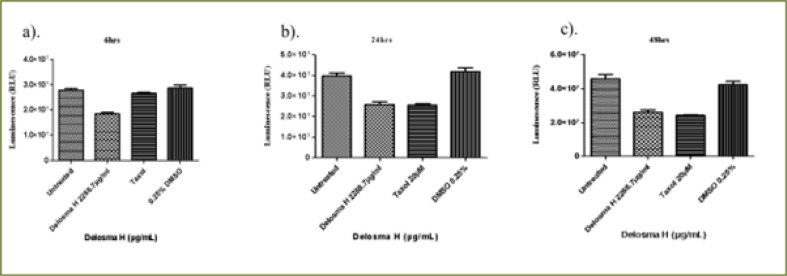
The changes in viability of THP-1 monocyte cells at (a) 6 hours (b) 24 hours (c) 48 hours incubation with an (IC50) value for Delosma H (2268.7µg/mL) and positive control (Taxol 20 µM)

As shown in Figure 2, *Delosma H* (2268.7 µg/mL) significantly reduced the viability of THP-1 cells at 6, 24 and 48 hours whereas, Taxol significantly reduced the viability at 24 and 48 hours when compared to the untreated cells (P < 0.0001). The effect of *Delosma H* was comparable to Taxol at 24 (64.9% versus 64.1%) and 48 hours (56.9% and 52.9%), respectively.

**Figure 3:**
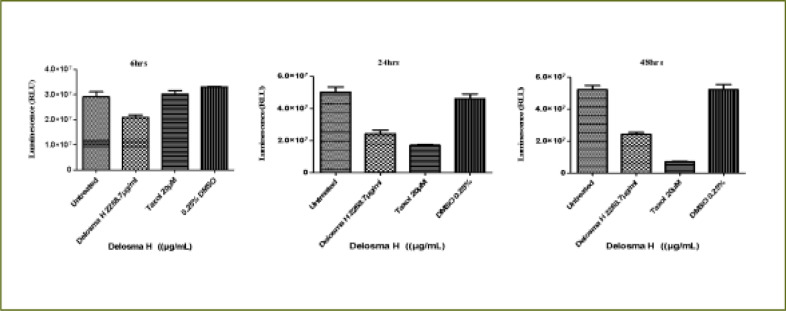
The changes in viability of Jurkat lymphocytes cells at (a) 6 hours (b) 24 hours (c) 48 hours incubation with an (IC50) for Delosma H (2268.7 µg/mL) and positive control (Taxol 20 µM)

Overall, after 6 hours of exposure to *Delosma H* and Taxol, the viability of Jurkat cells was significantly different between the treatment and the untreated groups (ANOVA p=0.0012, Figure 3). This was due to significantly lower cell viability in Delosma H treated (71.6%) compared to untreated cells (P < 0.05). Taxol (positive control) did not reduce cell viability at all. (Figure 3a). At 24 and 48 hours, *Delosma H* significantly reduced cell viability to 48.4% and 46.4%, respectively (P < 0.0001). The effect of Delosma H was comparable to Taxol which reduces cell viability to 34.1% and 13.8% at 24 and 48 hours, respectively (P < 0.0001, Figure 3).

### Mshikazi extract

**Figure 4:**
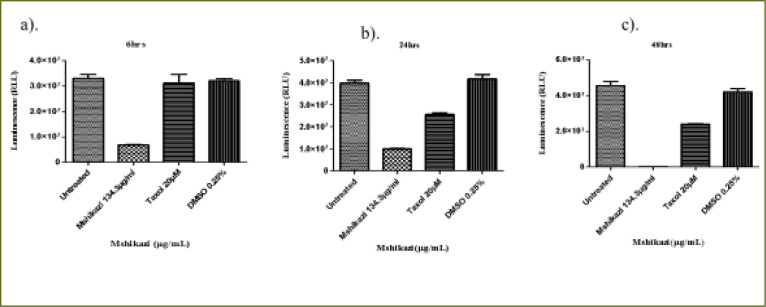
The changes in cell viability of THP-1 monocytes cells at (a) 6 hours (b) 24 hours (c) 48 hours incubation with an (IC50) value for Mshikazi (134.3µg/mL) and positive control (Taxol 20 µM)

At 6 hours of incubation, there was a significant difference in the viability of THP-1 cells treated with *Mshikazi* extract at (IC50) dose (134.3 µg/mL), Taxol (20 µM) and untreated cells (P < 0.0001, Figure 4a). *Mshikazi* extract significantly decreased the THP-1 viability to 20.5% compared to the positive control (Taxol, 94.2%) and the untreated THP-1 cells (Figure 4a). There was no significant difference between the untreated THP-1 cells and the treated positive control (Taxol). At 24 h, *Mshikazi* extract was still found to be very active, reducing the viability of THP-1 cells to 24.7%, better than the positive control at 64.1% albeit statistically significantly lower than the untreated THP-1cells (P < 0.0001, Figure 4b). Cell viability was significantly lower in *Mshikazi* extract treated and compared to Taxol treated cells (P < 0.005). At 48 h, both *Mshikazi* extract and Taxol statistically significantly reduced the viability of THP-1 cells compared to the untreated cells (P < 0.0001, Figure 4c). Cell viability was significantly lower in *Mshikazi* extract treated (0.5%) compared to Taxol treated (52.9%) cells.

**Figure 5:**
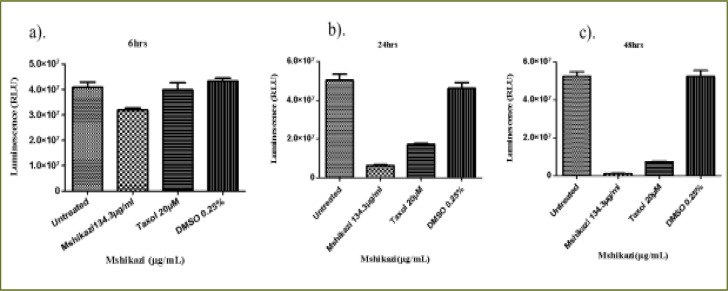
The changes in viability of Jurkat lymphocytes cells at (a) 6 hours (b) 24 hours (c) 48 hours incubation with an (IC50) value for Mshikazi (134.3µg/mL) and positive control (Taxol 20 µM)

The 6-hour treatment of the Jurkat lymphocyte cells with *Mshikazi* extract reduced its viability to 78.0% in relation to the untreated cells and Taxol (100 %, P < 0.0134, Figure 5a). At 24 hours (Figure 5b), there was a significant difference in the viability of Jurkat cells treated with *Mshikazi* extract compared to Taxol and untreated cells with (P < 0.0001). *Mshikazi* extract significantly reduced the viability of Jurkat cells to 12.4%, better than the positive control (34.1%). At 48 hours of treatment (Figure 5c), there was a significant difference in the viability of Jurkat cells treated with *Mshikazi* extract compared to Taxol and untreated cells with (P < 0.0001). The activity of *Mshikazi* extract increased significantly leading to the viability of Jurkat cells of 2.1%. The positive control (Taxol) also had a significant effect on the viability of Jurkat cells after 48 h (13.8%) but to a lesser extent compared to the *Mshikazi* extract (Figure 5).

### Emelia- M (EMB) extract

**Figure 6:**
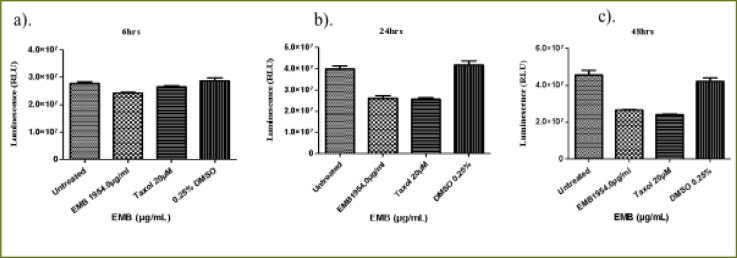
The changes in viability of THP-1 monocytes cells at (a) 6 hours (b) 24 hours (c) 48 hours incubation with an (IC50) value for Emelia M (EMB) (1954.0 µg/mL) and the positive control (Taxol 20 µM)

At 6 hours (Figure 6a), the EMB extract at (IC50) dose (1954.0 µg/mL) decreased THP-1 viability to 87.2% compared to the positive control (94.2%) (P < 0.0105). At 24 hours of treatment, EMB extract significantly reduced THP-1 cell viability compared to Taxol and untreated cell (P < 0.0001) (Figure 6b). There was a significant difference in the cell viability of Taxol (positive control) compared to the untreated cells. The effect of the 24 hours treatment with EMB extract (65.5%) showed no significant difference from that of the positive control (Taxol, 64.1%) in relation to the untreated THP-1cells (Figure 6b). At 48 hours of treatment, compared to the untreated cells (P < 0.0001, Figure 6c), EMB extract reduced the viability of THP-1 cells to 58.1% which was similar to what was observed for the positive control (52.9%). However, there was no significant difference in the viability of the THP-1 cells treated with EMB extract and Taxol (positive control).

**Figure 7:**
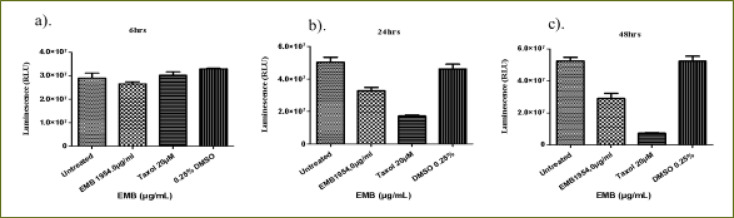
The changes in cell viability of Jurkat lymphocytes cells at (a) 6 h (b) 24 h (c) 48 h incubation with an (IC50) value for Emelia M (EMB) (1954.0µg/mL) and positive control (Taxol 20 µM)

The 6 hour treatment of Jurkat cells with EMB extract had a very low effect on the viability of the Jurkat cells (91.4%) compared to the untreated Jurkat cells (P < 0.0488, Figure 7a) whereas Taxol had no effect at all. At 24 h, there was a significant difference in the activity of EMB extract reducing the viability of Jurkat cells to 65.1% but with a lesser effect compared to the positive control (34.1%) relative to the untreated Jurkat cells (P < 0.0001, Figure 7b). At 48 hours, the EMB extract reduced the viability of Jurkat cells to 55.4% while Taxol reduced the viability of the Jurkat cells to 13.8% (P < 0.0001, Figure 7c).

### Activation of caspases 3/7 on THP-1 and Jurkat cells

The caspase 3/7 activity was used to determine the level of activation of apoptosis induced by the three extracts and positive control (Taxol) on THP-1 monocytes and Jurkat lymphocytes cells. The percentage values of the increase in the caspase activity of the two cancer cells after the treatment with the three extracts and Taxol that serves as positive control are shown in Table 2.

**Table 2:** The caspase 3/7 activity of the three extracts and Taxol on THP-1 monocytes and Jurkat lymphocytes cells at 24 and 48 hours

Traditional herbal	THP-1 cells (%)			Jurkat cells (%)		

medicine/Control	6 h	24 h	48 h	6 h	24 h	48 h
*Delosma H* (2268.7 µg/mL)		119.7	119.2		67.7	117
*Mshikazi* (134.3 µg/mL)	73.5	82.8	25.0	343.3	197.5	175
*Emelia- M* (1954 µg/mL)		387.5	180.0		156.4	400
Taxol (20 µM)	138.8	540.1	226.5	137.5	425.5	795

### Delosma H extract

**Figure S3:**
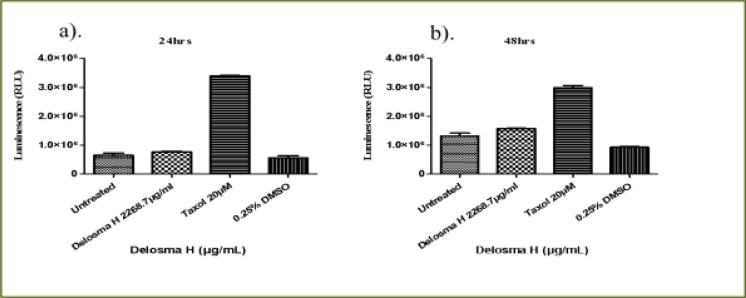
Caspase 3/7 activity of THP-1 monocyte for (a) 24 hours, (b) 48 hours incubation with (IC50) of Delomas H (2268.7 µg/mL) and positive control (Taxol 20 µM)

The THP-1 monocytes and Jurkat lymphocytes were treated with an (IC50) value of *Delosma H* (2268.7µg/mL) extract and positive control (Taxol 20 µM) at 24 and 48 hours. At 24 hours, *Delosma H* extract at IC50 dose increased the caspase activity to 119.7% and positive control (Taxol 20 µM) increased the caspase activity to 540.1% when compared to the untreated THP-1 cells (P < 0.0001) (Figure S3a). The same trend occurred at 48 h, where *Delosma H* extract at (IC50) dose increased the caspase 3/7 activity to 119.2% and positive control (Taxol 20 µM) increased caspase3/7 activity by 226.5% as compared to the untreated THP-1 cells with P < 0.0001 (Figure S3b).

**Figure S4:**
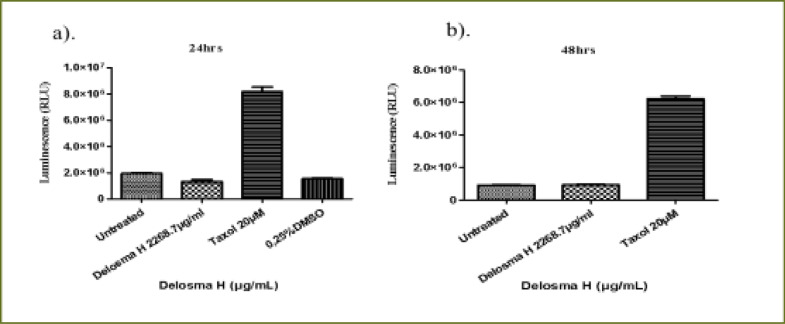
Caspase 3/7 activity of Jurkat lymphocytes for (a) 24 hours, (b) 48 hours incubation with (IC50) of Delomas H (2268.7 µg/mL) and positive control (Taxol 20 µM)

At 24 hours, *Delosma H* extract at (IC50) concentration decreased the caspase 3/7 activity of Jurkat cells (67.7%) as compared to the untreated Jurkat cells with P < 0.0001. The positive control significantly increased caspase 3/7 activity of Jurkat cells (425.5%) (Figure S4a). At 48 hours, *Delosma H* extract increased caspase 3/7 activity of Jurkat cell to 117% but this was not statistically significant compared to untreated cells. On the contrary, Taxol significantly increased caspase 3/7 activity (795%) when compared to the untreated Jurkat cells (P < 0.0001, Figure S4b).

### Mshikazi extract

**Figure S5:**
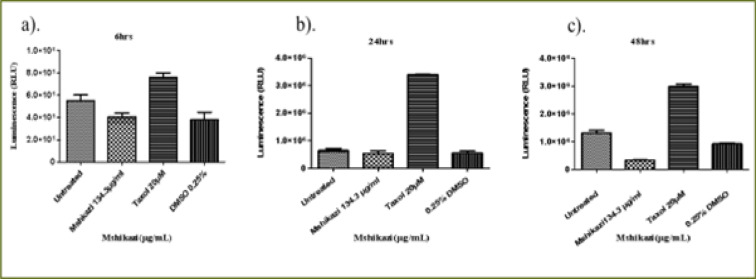
Caspase 3/7 activity of THP-1 monocyte for (a) 6 h (b) 24 hours (c) 48 hours incubation with (IC50)of Mshikazi (134.3 µg/mL) and positive control (Taxol 20 µM)

The THP-1 monocytes and Jurkat lymphocytes were treated with an (IC50) value of *Mshikazi* (134.3µg/mL) extract of THM and the positive control (Taxol 20 µM) at 6, 24 and 48 hours. At 6 hours, there was a decrease in caspase 3/7 activity of THP-1 cells when treated with *Mshikazi* extract (73.5 %), whereas there was an increase when treatment with the positive control (138.8 %) as compared to untreated cells (P < 0.0023, Figure S5a). At 24 hours, *Mshikazi* reduced caspase 3/7 activity to 82.8% as compared to the untreated cells and Taxol (P < 0.0001, Figure S5b). A further decrease in the caspase 3/7 activity was observed at 48 hours of treatment of THP-1 cells with *Mshikazi* extract (25.0%) when compared to the untreated cells and the positive control Taxol (p < 0.0001, Figure S5c). On the contrary, Taxol significantly increased caspase activity at 6, 24 and 48 hours of exposure.

**Figure S6:**
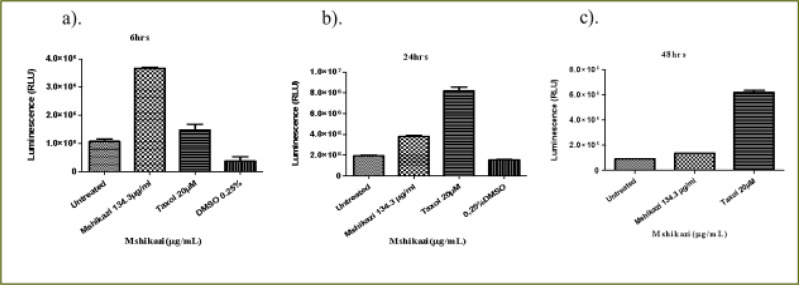
Caspase 3/7 activity of Jurkat lymphocytes for (a) 6 h (b) 24 hours (c) 48 hours incubation with (IC50) of Mshikazi (134.3 µg/mL) and positive control (Taxol 20 µM)

At 6 hours treatment of the Jurkat cells with 134.3 µg/mL concentration of *Mshikazi* extract lead to a significant increase in the caspase 3/7 activity (343.3 %) as compared to a positive control (137.5 %) (P < 0.0001, Figure S6a). At 24 h of treatment with *Mshikazi* extract, the caspase 3/7 activity (197.5%) remained significantly higher than untreated cells while that of positive control increased more significantly to 425.5% in comparison with the untreated cells (P < 0.0001, Figure S6b). At 48 hours, the effect of *Mshikazi* extract was similar (175%) while that of Taxol further increased caspase activity to 795% when compared to the untreated cells (P < 0.0001, Figure S6c).

### Emelia- M (EMB)

**Figure S7:**
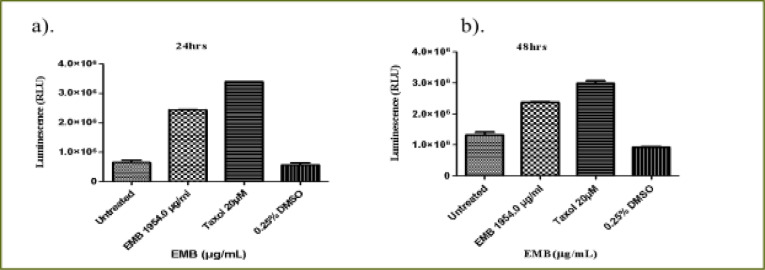
Caspase 3/7 activity of THP-1 monocyte for (a) 24 hours, (b) 48 hours incubation with EMB (1954.0 µg/mL) and positive control (Taxol 20 µM)

**Figure S8:**
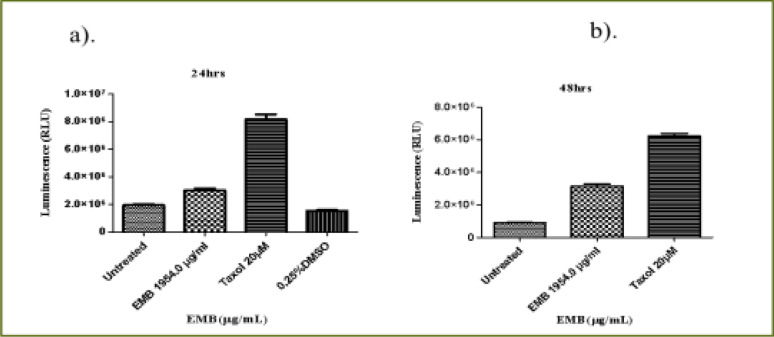
Caspase 3/7 activity of Jurkat lymphocytes for (a) 24 hours, (b) 48 hours incubation with EMB (1954.0 µg/mL) and positive control (Taxol 20 µM)

The THP-1 monocytes and Jurkat lymphocytes were treated with an (IC50) value of EMB (1954.0 µg/mL) extract of THM and the positive control (Taxol 20 µM) at 24 and 48 hours. EMB extract and positive control increased the activity of caspase 3/7 of THP-1 cells at 24h to 387.5% and 540.1%, respectively when compared to the untreated THP-1 cells with P < 0.0001 (Figure S7a). The positive control gave a higher increase in the caspase 3/7 activity compared to that EMB extract (P = 0.005). At 48 h, the increase in the caspase 3/7 activity from EMB extract was 180% while that of control was 226.5% in relation to the untreated cell (P < 0.0001, Figure S7b). Both EMB extract and positive control increased the caspase 3/7 activity of Jurkat cells at 24 hours as compared with the untreated Jurkat cells (P < 0.0001) but a higher activity was observed in the positive control (425.5%) compared to the EMB extract (156.4%) as shown in Figure S8a. At 48 hours of treatment, EMB extract increased the caspase 3/7 activity to 400% when the positive control also increased the caspase activity to 795% when compared to the untreated cells (P < 0.0001, Figure S8b).

### Caspase 8 activity on THP-1 and Jurkat cells

Caspase 8 activity of THP-1 monocytes and Jurkat lymphocytes cells was determined at 6 hours of incubation with (IC50) of *Delosma H* (2268.7 µg/mL), *Mshikazi*, (134.3 µg/mL) and EMB (1954.0 µg/mL) extracts and positive control (Taxo,l 20 µM). The percentage values of the caspase 8 activity for the treatment of the two cancer cells for 6 hours are reported in Table 3 for the three extracts.

**Table 3:** The percentage of caspase 8 activity of the three traditional herbal medicines and Taxol on THP-1 monocytes and Jurkat lymphocytes cells at 6 hours

Traditional herbal medicines / Control	THP-1 monocyte cells 6 hours (%)	Jurkat lymphocyte cells 6 hours (%)
*Delosma H* (2268.7µg/mL)	107.9	79.7
*Mshikazi*, (134.3 µg/mL)	29.4	104.4
*Emelia-M* (1954.0 µg/mL)	98.7	62.8
Taxol (20 µM)	142.3	111.7
0.25% DMSO	170.4	77.4

### Delosma H extract

**Figure S9:**
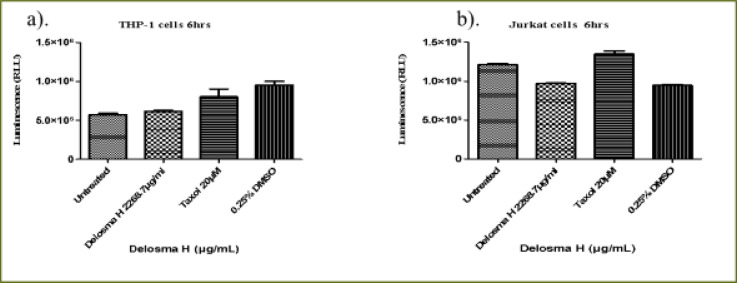
Caspase 8 activity on (a) THP-1 monocyte and (b) Jurkat lymphocyte cells after 6 hours incubation with (IC50) of Delosma H extracts (2268.7 µg/mL) and positive control (Taxol 20 µM)

*Delosma H* extract and positive control at 6 hours, increased caspase 8 activity of THP-1 cells compared with the untreated THP-1 cells with a P-value of 0.0082 (Figure S9a). The positive control leads to a higher increase in the activity of caspase 8 (142.3%) compared to the *Delosma H* extract (107.9%). Treatment of Jurkat cells with *Delosma H* extract led to a decrease in the activity of caspase 8 (79.7 %) while positive control still leads to an increase in the caspase 8 activity (111.7 %) when compared to the untreated Jurkat cells (P < 0.0001, Figure S9b).

### Mshikazi extract

**Figure S10:**
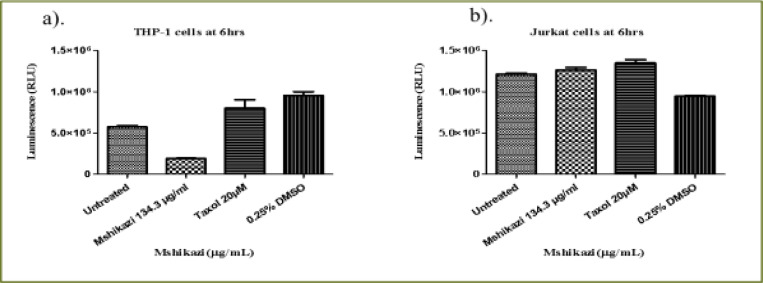
Caspase 8 activity on (a) THP-1 monocyte and (b) Jurkat lymphocyte cells after 6 hours incubation with (IC50) Mshikazi, (134.3 µg/mL) and positive control (Taxol 20 µM)

The 6 hours treatment of THP-1 cells with *Mshikazi* extract caused a significant decrease in the activity of caspase 8 (29.4%) while the positive control leads to an increase in the caspase 8 (142.3%) when compared to the untreated THP-1 cells (P < 0.0001, Figure S10a). On the other hand, the 6 hours treatment of the Jurkat cells with Mshikazi extract resulted in an increase in the caspase 8 activity (104.4%) just as a positive control (111.7 %) when compared to the untreated cells (P < 0.0001, Figure S10b).

### Emelia- M (EMB)

**Figure S11:**
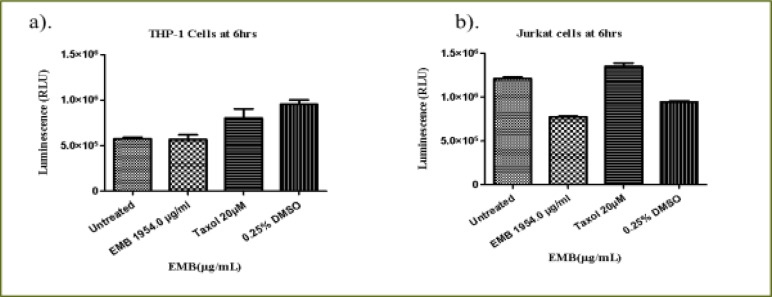
Effect of caspase 8 activity on (a) THP-1 monocyte and (b) Jurkat lymphocyte cells after 6 hours incubation with (IC50) of EMB (1954.0 µg/mL) and positive control (Taxol 20 µM)

The 6 hours of treatment of THP-1 cells with EMB extract caused a slight decrease in caspase 8 activity of THP-1 cells (98.7%) while positive control leads an increase (142.3 %) as compared to the untreated cells with (P < 0.006, Figure S11a). The 6 hours treatment of Jurkat cells with EMB extract lead to a significant decrease in caspase 8 activity (62.8%) while that of the positive control leads to an increase in caspase 8 activity (111.7%) as compared to untreated cells (P < 0.0001, Figure S11b).

### DNA fragmentation assay - Hoechst 33342 staining/propidium iodide

The apoptotic cells were determined by observing the variations in the nucleus morphology of incubated cells using Hoechst staining/propidium iodide dye (26). THP-1 monocytes (1 × 10^7^ cells) and Jurkat lymphocytes (1 × 10^7^ cells) were treated with EMB (1954.0 µg/mL), *Mshikazi*, (134.3 µg/ml), *Delosma H* (2268.7 µg/mL), 20µM Taxol (positive control) and a solvent control (0.25% DMSO) and incubated for 24 and 48 hours.

**Figure 8:**
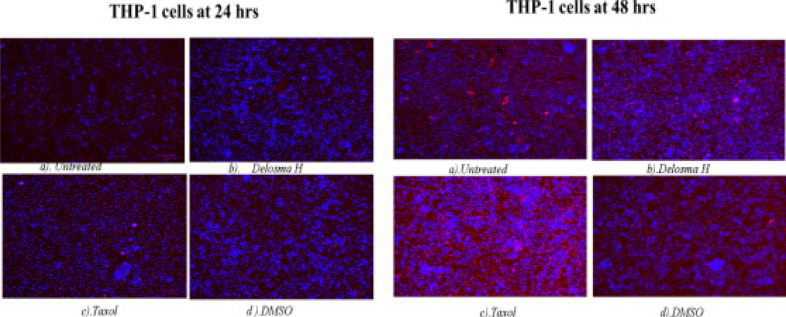
Hoechst/ propidium iodide staining of THP-1 cells at 24 and 48 hours of incubation. (a) Untreated, (b) Delosma H, (c) Taxol and (d) DMSO

At 24 hours, exposure of THP-1 monocytes to Delosma H extract resulted in many early apoptotic cells (deep blue fluorescent). Delosma H extract induced DNA fragmentation and apoptotic body formation with deep blue colour (Hoechst staining) (Figure 8) At 48 h, Delosma H and Taxol (positive control) induced early apoptotic cells (deep blue fluorescent) but Delosma H extract also showed many necrotic (fainting red fluorescent). Taxol induced late apoptotic with cells showing fragmented nuclei (red fluorescent). Both *Delosma H* and Taxol induced irregular shape, membrane blebbing, and apoptotic body formation in THP-1 monocytes as compared to the untreated cells.

**Figure 9:**
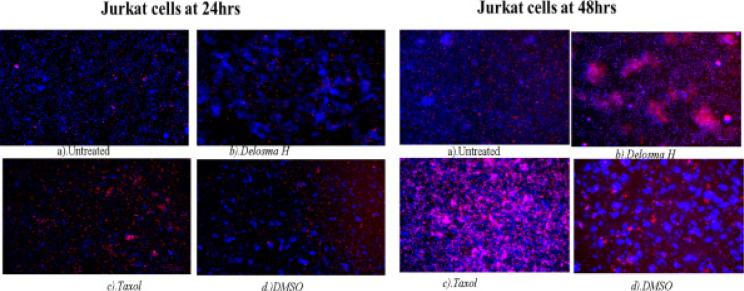
Hoechst/ propidium iodide staining of Jurkat cells at 24 and 48 hours of incubation. (a) Untreated, (b) Delosma H, (c) Taxol and (d) DMSO

At 24 hours exposure of Jurkat lymphocyte cells to Delosma H extract resulted in an early apoptotic cell (deep blue fluorescent) while Taxol shows more late apoptotic cells (red fluorescent). At 48 hours, Delosma H and Taxol are predominantly characterised with late apoptotic cells (red fluorescent) while Taxol is predominantly characterised by necrosis cells compared to the untreated cells that were still characterised by many cells with intact and round nuclei (Figure 9).

**Figure S12:**
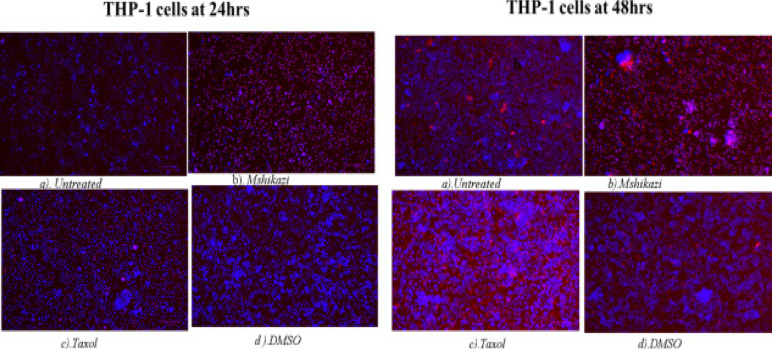
Hoechst/ propidium iodide staining of THP-1 cells at 24 hours and 48 hours of incubation. (a) Untreated, (b) Mshikazi (c) Taxol and (d) DMSO

**Figure S13:**
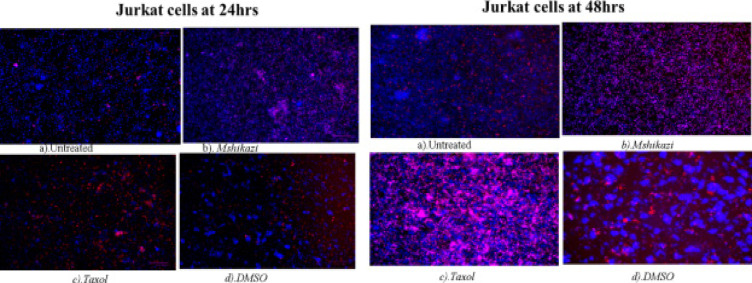
Hoechst/ propidium iodide staining of Jurkat cells at 24 hours and 48 hours of incubation. (a) Untreated, (b) Mshikazi (c) Taxol and (d) DMSO

The treatment of THP-1 cells with *Mshikazi* extract at 24 hours resulted in more late apoptotic cells and necrosis cells while positive control has some features of early apoptotic cells. At 48 hours, *Mshikazi* extract is still more of late apoptotic cells with fragmented nuclei and also has some level of necrosis cells. The positive control at 48hours is characterised by both early apoptotic cells and late apoptotic cells with fragmented nuclei (Figure S12). At 24 hours of treatment of Jurkat cells with *Mshikazi* extract, the morphology is more of early apoptotic cells while the positive control is more of late apoptosis with fragmented nuclei. By 48 hours of treatment, the *Mshikazi* extract showed more of both apoptotic and necrosis cells but the necrosis cells are more obvious in the positive control (Figure S13).

**Figure S14:**
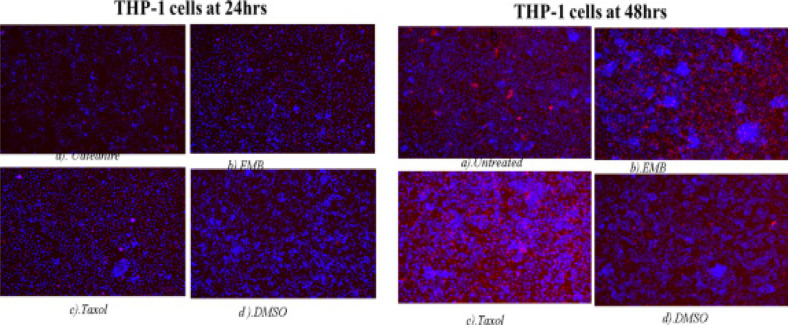
Hoechst/ propidium iodide staining of THP-1 cells at 24 hours and 48 hours of incubation. (a) Untreated, (b) EMB (c) Taxol and (d) DMSO

**Figure S15:**
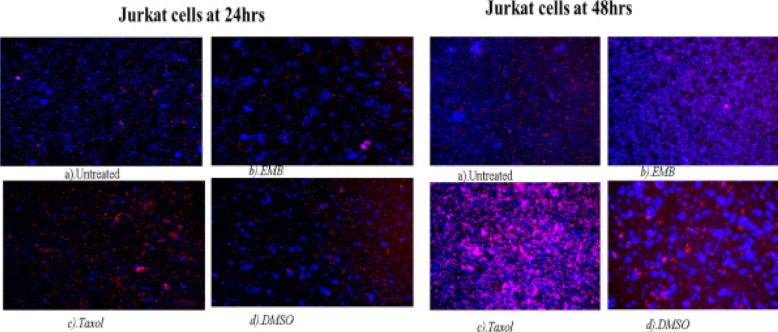
Hoechst/ propidium iodide staining of Jurkat cells at 24 hours and 48 hours of incubation. (a) Untreated, (b) EMB (c) Taxol and (d) DMSO

The 24 hours of treatment of THP-1 cells with EMB extract and positive control shows many early apoptotic cells. At 48 hours, EMB extract is still characterised by many early apoptotic cells with many irregular shapes and membrane blebbing and also some late apoptotic cells with DNA fragmentation. The positive control at 48h is characterised predominantly by late apoptotic cells with DNA fragmentation than the early apoptotic cells (Figure S14). At 24 hours of treatment of Jurkat lymphocyte cells with EMB extract showed more early apoptotic with many irregular shapes and membrane blebbing while positive control shows more late apoptotic cells with DNA fragmentation. The morphology of the Jurkat cells at 48 hours of EMB extract treatment is characterised predominantly by early apoptotic cells while the positive control is more of late apoptotic and some necrosis and early apoptotic cells (Figure S15).

### Flow cytometry

**Figure 10:**
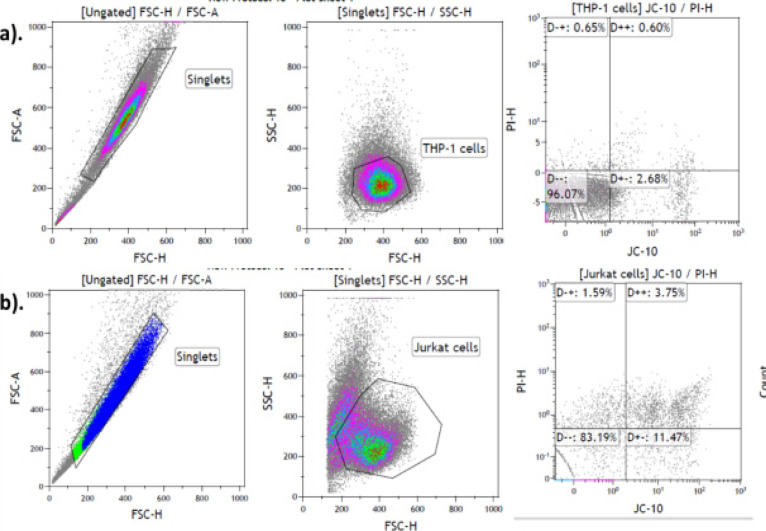
Gate strategy for the flow analysis of EMB, Mshikazi and Delosma H on THP-1 and Jurkat cells

Flow cytometry was used to determine the type of cell death induced by EMB, *Mshikazi* and *Delosma H* extracts on THP-1 and Jurkat cells (Figure 10). Over 24 hours of incubation of THP-1 monocytes and Jurkat lymphocytes cells, *Mshikazi* extract induced the most significant level of late apoptosis in THP-1 monocytes and early apoptosis in Jurkat lymphocytes cells compared to untreated cells and Taxol. *Mshikazi* had a minimal necrotic effect on THP-1 cells (Table 4).

**Table 4:** Apoptosis, necrosis and Live THP-1 and Jurkat cells in a sample treated with EMB, *Mshikazi* and *Delosma H* THMs over 24 hours of the incubation period

THP-1 CELLS (%)					JURKAT CELLS (%)			

Sample type	Live cells	Early apoptosis	Late apoptosis	Necrosis	Live cells	Early apoptosis	Late apoptosis	Necrosis
*Untreated*	93.51	5.35	0.78	0.36	79.29	15.47	3.71	1.54
EMB	70.58	14.15	8.58	6.69	66.58	25.99	6.49	0.93
*Mshikazi*	32.95	15.04	41.57	10.44	50.49	44.57	2.98	1.96
*Delosma H*	70.76	14.37	10.20	4.47	53.07	33.82	12.57	0.73
*Taxol*	80.68	10.00	5.83	3.50	48.77	42.86	6.93	1.44
*0.25%DMSO*	87.66	6.00	3.06	3.28	78.64	17.43	3.06	0.87

At 24 hours, EMB and Delosma H extracts induced apoptosis in THP-1 monocytes whereas the Taxol induced more apoptosis in Jurkat cells. The order of effectiveness in inducing apoptosis for the three extracts were in the order *Mshikazi > Delosma H >* EMB.

### Liquid chromatography-mass spectrophotometry (LC-MS)

**Figure 11:**
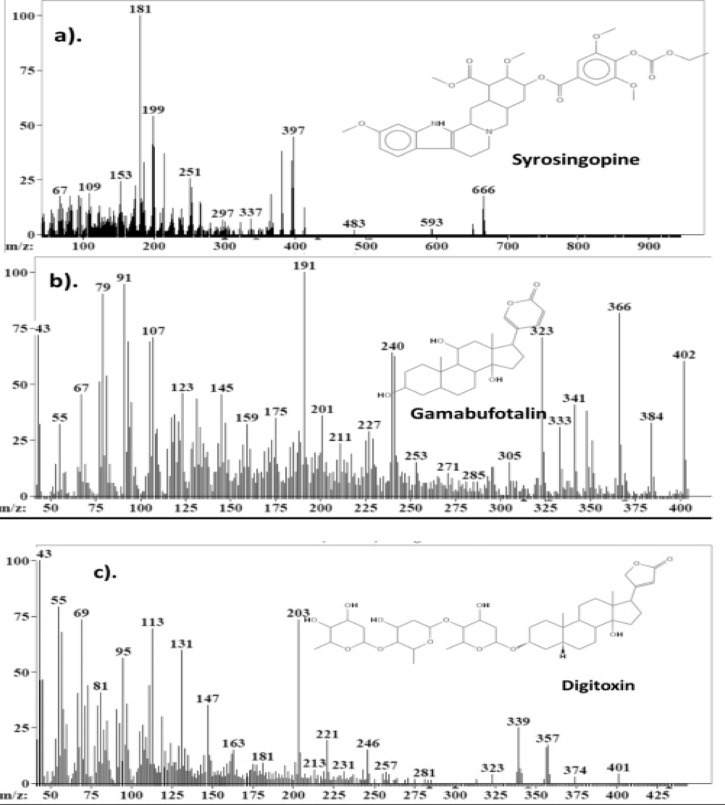
Mass spectra of a) syrosingopine in EMB and Delosma H, b) gamabufotalin in Mshikazi and Delosma H and c) digitoxin in EMB and Mshikazi traditional herbal medicines

The chemical compounds present in EMB, Mshikazi and Delosma H were determined using LC-MS. These compounds were identified through the mass spectrophotometer of the LC in both positive and negative scanning processes. The results are presented in Supplementary Tables S1, S2, and S3. The LC-MS data were analysed and matching with predicted molecules was done using the AMDIS version 2.73 package obtained from NIST 25 (Figure 11).

**Table S1:** Chemical compounds identified in the aqueous EMB traditional herbal medicine

*Sample*	*Retention time*	*Name of compound*
**1**	8.8443	2,6-Bis (phenyl methylene)-cyclohexanone
**2**	9.4774	Hydrocortisone acetate
**3**	9.9775	Picene
**4**	10.1	Diphenyl ester phosphoramidic acid
**5**	11.0141	Octicizer
**6**	12.2653	9-Bromo-9H-fluorene
**7**	12.3843	Hydroxylupanine
**8**	12.9104	Colchicine
**9**	16.3781	1,2-Dihydro-3-methyl-benz[j]aceanthrylen-1-ol
**10**	16.8809	Syrosingopine
**11**	17.5199	pentachloro-benzenethiol
**12**	18.2868	Cortisone
**13**	18.4004	Retinol
**14**	18.6277	Digitoxin
**15**	18.7458	Beclomethasone
**16**	19.9052	Pentachloronitrobenzene
**17**	21.1698	11 -α-Hydroxy-17 α-methyltestosterone
**18**	21.58	4-Dimethyl-3-oxo-methylester-vobassan-17-oic acid
**19**	21.7028	1,2-ethanediylbis[trichloro]-silane
**20**	32.6311	Iodoquinol
**21**	38.1383	Hexachloroacetone
**22**	11.5042	β-Carotene
**23**	13.5899	Dienochlor
**24**	14.2249	Hydrochlorothiazide
**25**	17.1712	Methoserpidine
**26**	17.563	tert-Butyl 2,4,5-trichlorophenylcarbonate
**27**	17.68	1,2-Dimethyl-1,1,2,2-tetraphenyldisilane
**28**	18.2687	Ricinoleic acid
**39**	19.5627	Rescinnamine
**30**	28.9478	(Acetyloxy)triphenylstannane
**31**	28.9768	Cholecalciferol

**Table S2:** Chemical compounds identified in the aqueous *Mshikazi* traditional herbal medicine

*Sample*	*Retention time*	*Name of compound*
1	2.6748	4-Hydroxy-n-heptylesterbenzoic acid
2	9.5887	Phenyl-diphenyl ester phosphoramidic acid
3	10.3217	Dibenzylketoxime
4	11.1697	Glycerol-1-palmitate
5	11.691	Tetraphenyl-germane
6	12.443	1,2-Benzenedicarboxylic acid, dinonyl ester
7	12.8388	Gamabufotalin
8	12.9594	2,6-Bis(phenylmethylene)-cyclohexanone
9	13.5017	Cyclothiazide
10	14.0286	(Triphenylphosphoranylidene)acetaldehyde
11	14.1461	Picene
12	14.6824	Bisacodyl
12	14.8026	Warfarin
14	15.2235	N-Benzyloxycarbonyl-L-tyrosine
15	15.8839	Kepone
16	16.4214	(+)-Colchicine
17	16.9452	9-Chloroacridine
18	17.701	Daniquidone
19	17.7	4′-Pentyl-[1,1′-biphenyl]-4-carbonitrile
20	18.2224	Tetraphenylhydrazine
21	18.4625	12,13-Dihydro-7H-dibenzo(a,g)carbazole
22	19.4491	Methylestereicosanoic acid
23	19.9893	Disperse Red 11
24	20.6362	Estra-1,3,5(10)-trien-3-ol
25	20.7583	Vincamine
26	21.3778	tris(3-methylphenyl) ester phosphoric acid
27	21.781	Tetrachlorvinphos
28	22.3067	9,10-Dihydro-9,9-dimethylacridine
29	29.3287	Tetrabutylstannane
30	29.8226	Trihexyl ester boric acid
31	30.438	Sulfathiazole
32	30.4565	Cortisone
33	31.09	Pentachlorobenzene
34	31.4952	2,3-Dibromo-1-propanol, phosphate (3:1)
35	32.0417	Octabenzone
36	32.811	Hydrochlorothiazide
37	33.2148	Digitoxin
38	33.4458	Testosterone enanthate
39	33.5649	Isopropyl palmitate
40	34.033	Ergocalciferol
41	34.679	Lincomycin
42	37.5856c	Iodoquinol

**Table S3:** Chemical compounds from the aqueous *Delosma* H traditional herbal medicine

*Sample*	*Retention time*	*Name of compounds*
1	1.5879	11H-Benzo[a]carbazole
2	1.9808	Gamabufotalin
3	2.0973	Trihexyl ester boric acid
4	7.9885	Sucrose Octaacetate
5	8.0603	3-Iodo-phenol
6	8.1449	Prednisolone acetate
7	9.8596	3-β-Cholesta-5,7-dien-3-ol-acetate
8	10.8001	Methoserpidine
9	14.7803	β-Carotene
10	16.0088	Testosterone acetate
11	16.5709	2,3-Dibromo-1,4-butanediol
12	17.2678	Hydrocortisone
12	17.3027	Retinol
14	17.3798	1,3,5-tribromo-2-methoxy-benzene
15	17.696	Syrosingopine
16	17.777	1,3,5-tribromo-2-methoxy-benzene
17	17.8908	Phenomorphan
18	18.0018	Cortisone
19	18.5006	Hydrocortisone acetate
20	19.1813	1,3,5-Tribromo-2-methoxy-benzene
21	24.4145	2,6-bis(phenylmethylene)-cyclohexanone
22	29.3185	3,4-Dihydro-2-methoxy-2-methyl-4-phenyl-2H,5H-pyrano[3,2-c][1] benzopyran-5-one
23	30.1836	Disperse Red 11
24	30.4773	Sulfathiazole
25	30.6942	Trihexyl ester boric acid
26	30.9252	Tetrabutylstannane
27	31.4529	Bufotalin
28	31.8565	Lauryl gallate
29	32.0777	2,4,6-Tribromobenzenamine
30	33.2465	Mazindol
31	33.8687	N-Phenyl-4-(phenylazo)benzenamine
32	33.9105	Testosterone enanthate
33	34.4363	4-Dimethyl-3-oxo-methyl ester vobassan-17-oic acid
34	34.9458	Ethisterone
35	35.4318	1,2,3,4-Tetrachloro-5,5-dimethoxy-1,3-cyclopentadiene
36	35.7557	Hexachloroacetone
37	35.8182	Iodoquinol
38	37.476	3-Methylbenz[j]aceanthrylene

## Discussion

Medicinal plants have been used for ages to combat cancer and over 3000 plants have been reported to cause cytotoxicity of cancer cells, globally 27. Almost 80% of rural Africans use traditional medicine for their primary health care needs and medicinal plants form a major part of traditional medicine 28. Many of these African medicinal plants have been tested for their cytotoxic potential. In our research, three traditional herbal medicines, namely, EMB, *Mshikazi* and *Delosma H* that have been used by African traditional healers for the treatment of cancer were evaluated for their anticancer activity. Their effects on cell viability were examined using normal human peripheral blood mononuclear cells (PBMCs) and the half-maximal inhibitory concentration (IC50) was used to screen the cytotoxic effect on THP-1 monocyte cells and Jurkat lymphocyte cells in vitro. In addition, the mechanisms by which they induced apoptosis were assessed. At 24 and 48 hours, the EMB, *Mshikazi* and *Delosma H* extracts decreased the viability of PBMCs as the concentration increased with time and IC50 values were established.

The IC50s suggest that three extracts have low toxicity toward PBMCs. This finding is in agreement with a report on the medicinal plant, *Centella asiatica*, which showed a significant decrease in the viability of PBMCs at 24 and 72 hours 29. Moreover, Sudeep, Nithya and Kiranmayee 30 reported that Annona squamosa, Datura metel, and Mentha piperita extracts were toxic to cultured lymphocytes (PBMCs) from humans. *Annona squamosa* showed the ability to inhibit cell survival compared to the other two extracts. These findings can be related to our results where the *Mshikazi* extract resulted in the lowest viability of PBMC cells compared to the other two herbal medicines.

The (IC50) of EMB, *Mshikazi* and *Delosma H* extracts, and Taxol (positive control) were used to evaluate the anticancer effect on THP-1 and Jurkat cells at 6, 24 and 48 hours. The results clearly showed that EMB, *Mshikazi* and *Delosma H* extracts inhibit the growth of THP-1 monocyte and Jurkat lymphocytes cells.

The three extracts significantly inhibited cell growth of THP-1 at 6, 24 and 48hours while Taxol only began to have an effect at 24 hours. The effect of *Delosma H* and EMB was similar to Taxol at 24 and 48hours while *Mshikazi* had the highest effect.

At 6, 24 and 48 hours of treatment, the three extracts significantly inhibited cell growth of Jurkat while Taxol only began to have an effect at 24 hours. The effect of *Delosma H* and EMB was similar to Taxol at 24 and 48hours while *Mshikazi* had the highest effect.

Studies have shown plant extracts inhibit cell growth in THP-1 monocytes and Jurkat lymphocytes cells. For instance, the *T. welwitschii* plant extract at a low concentration of 31.25 µg/mL inhibited the growth of Jurkat T cells 31 and *Centella asiatica* at 0.2–0.8 mg/mL significantly decreased the cell viability of THP-1 cells 29. The *Mshikazi* extract at a low IC50 concentration (134.3 µg/mL) showed a greater inhibitory effect on the cell proliferation for THP-1 monocytes and Jurkat lymphocytes cells, even at a 6 h incubation period. The *Mshikazi* extract was more effective than the chemotherapy drug Taxol which was used as the positive control. In agreement with our findings is the literature report on *Tulbaghia violacea* Harv leaf (TVL) extracts, which exhibited inhibitory effects on T-lymphocyte Jurkat cells 32 similar to this study for *Mshikazi* traditional herbal medicine. In addition, in agreement is another report on the cytotoxicity of crude fractions A to F of *Ageratum conyzoides* extract on Jurkat lymphocytes cells (33), which significantly reduced the viability of Jurkat cells (P < 0.001). However, according to the report, the subfractions (1, 2, 4 to 6) obtained from further purification of fraction D of *Ageratum conyzoides* did not have any effect on the viability of Jurkat lymphocyte cells after treatment with 0 -100 µg/mL for 72 hours. This is not the case in our study because, after 48 h of treatment, all three extracts of traditional herbal medicines reduced the cell viability of Jurkat cells. At this same time of incubation period, the ATP level of the THP-1 monocyte cells and Jurkat lymphocyte revealed that EMB, *Mshikazi* and *Delosma H* extracts retained their activities for longer periods as evident in the significant decrease in cell viability.

Apoptosis can be induced by both intrinsic and extrinsic pathways which include a caspase cascade that acts through initiator caspases and executioner caspases. The chosen caspases were the initiator caspase-8 for the extrinsic pathway and the executioner caspase -3/7 34. When the initiator caspase-8 is triggered by apoptotic stimuli, then the active caspase-8 will initiate apoptosis by direct cleaving and activating executioner caspase-3/7 or activate the intrinsic apoptotic pathway to instigate apoptosis 35. There was an increase in caspase 3/7 activity from the treatment of the THP-1 cells with the Taxol which significantly increased activity from 138.8% in 6 h to 540.1% in 24 hours but dropped at 48 h to 226.5%. The same trend was observed with the EMB extract which was first characterised by a high increase iactivity of the caspase 3/7 at 24 h (387.5%) but dropped to 180% after 48 hours. The treatment with *Delosma H* extract resulted in a small increase in caspase 3/7 activity at 24 hours (119.7%) with no significant change by 48 hours (119.2%). However, for the *Mshikazi* extract treatment of THP-1 cells from 6, 24 and 48 hours, a consistent decrease in caspase 3/7 activity was observed (73.5%, 82.8% to 25%). The order of caspase 3/7 activity at the end of 48 h of treatment of the THP-1 cells with the three extracts and the positive control (Taxol) is Taxol > EMB > *Delosma H >> Mshikazi*.

The *Mshikazi* extract and Taxol significantly increased caspase 3/7 activity of Jurkat cells at 6, 24 and 48hours. EMB extracts increased the caspase 3/7 activity of Jurkat cells at 24 and 48h while the increase in caspase 3/7 of *Delosma H* only began to affect 48hour. The effect of EMB was similar to Taxol at 24 while *Mshikazi* had the highest effect at 6 hours.

The study on the caspase 3/7 activity of *Centella asiatica* extract was previously reported 29. *Centella asiatica* decreased caspase 3/7 activity of THP-1 over a concentration of 0.2–0.4 mg/mL but increased over a concentration of 0.05 and 0.8 mg/mL of *Centella asiatica* extract (24 h, P < 0.0001). This finding is similar to our findings where there was a decrease in the caspase3/7 activity of the *Mshikazi* extract on THP-1 cells and an increase in the caspase 3/7 activity of EMB and *Delosma H* extracts on THP-1 cells at 24 h (P < 0.0001). Belayachi 36 also reported that the hexane extract from *Retama monosperma* and Doxorubicin (1 µM), a positive control, significantly increased caspase 3/7 activity on Jurkat cells over 48 hours, similar to our findings. The EMB, *Mshikazi* and *Delosma H* extracts and Taxol (positive control) increased the caspase 3/7 activity of the Jurkat cells. Another study 37 reported that water-soluble polysaccharide extracts from Inonotus taiwanensis induced apoptosis through a caspase-independent pathway. The extract was found to decrease mitochondrial membrane potential and activated caspase-9 and caspase-3 on THP-1 and U937 cells.

The Taxol treatment of THP-1 cells increased caspase 8 activity (142.3%) at the end of 6 hours of treatment. The result from the *Delosma H* extract showed a slight increase in caspase 8 activity (107.9%) while the *Mshikazi* extract resulted in a significantly lower caspase 8 activity (29.4%) and also that of EMB extract resulted in a decrease in the caspase 8 activity (98.7%). The order of increasing caspase 8 activity after 6 hours of treatment of THP-1 cells was Taxol > *Delosma H* > EMB > *Mshikazi*.

The positive control treatment of Jurkat cells resulted in a slight increase in caspase 8 activity (111.7%). The *Mshikazi* extract also resulted in a slight increase in the caspase 8 activity (104.4%) but the result from *Delosma H* extract showed a significant decrease in the caspase 8 activity (79.7 %) and also that of EMB extract resulted in a decrease in caspase 8 activity (62.8%). The order of increasing caspase 8 activity after the 6 hours of treatment of Jurkat cells was Taxol > *Mshikazi* > *Delosma H* > EMB.

A study by Naidoo et al 29 reported that *Centella asiatica* increases caspase 8 activity of THP-1(0.05–0.8 mg/mL) at 24 hours but decreased caspase 8 activity a72 hours. This finding also corroborates our results of THP-1 cells treated with traditional herbal medicines with an increase in caspase 8 when treated with *Delosma H* but a decrease with EMB and *Mshikazi*. From our results, we see that only *Delosma H* extracts initiate apoptosis through the extrinsic pathway as a result of the increase in caspase 8 activity. All the extracts except Mshikazi induced apoptosis through increasing caspase 3/7 activation of THP-1. The three extracts induced better apoptosis in Jurkat cells than in THP-1 cells as evident from the higher level of caspase 3/7 activities.

The apoptosis morphological changes of the THP-1 monocytes and Jurkat lymphocytes were evaluated using *Mshikazi* (134.3 µg/mL), EMB (1954.0 µg/mL), *Delosma H* (2268.7 µg/mL) extracts and 20 µM Taxol (positive control). The results show that the untreated THP-1 and Jurkat cells show intact round nuclei. However, the apoptotic nuclei of cells treated with Mshikazi, EMB, Delosma H and Taxol (positive control) showed a morphological characteristic such as cell shrinkage, DNA damage, apoptotic body formation and membrane blebbing with heterogeneous staining (Hoechst/propidium iodide).

The morphology of Taxol-treated THP-1 cells using Hoechst staining/propidium iodide dye showed some early apoptosis at 24 hours which is maintained till 48 hours though it is also characterised by many late apoptotic cells. The *Delosma H* extract resulted in more early apoptotic cells at 24 hours than the positive control and at 48 h they are still more early apoptotic cells. After 24 and 48 hours of treatment with the *Mshikazi* extract, later apoptotic cells with DNA fragmentation and necrotic cells resulted. The 24 and 48 hours treatment with EMB extract was predominantly characterised by early apoptotic cells which were more pronounced at 48 hours with irregular shapes and membrane blebbing and some late apoptotic cells.

In Jurkat cells, the positive control showed more late apoptotic cells (red fluorescent) at both 24 and 48 hours with some level of necrotic cells at 48 hours. The *Delosma H* extract resulted in more early apoptotic cells at 24 hours but at 48 hours they were more late apoptotic cells with fragmented nuclei. The 24 hours treatment with *Mshikazi* extract was more for early apoptotic cells while 48 h was more for late apoptotic cells with DNA fragmentation and necrosis cells. At both 24 and 48 hours of treatment with the EMB extract, the morphology was predominantly characterised by early apoptotic cells.

These morphological changes in THP-1 and Jurkat cells are evidence that the three extracts induced apoptosis. The membrane blebbing shown by the extracts is an indication that the extracts of traditional herbal medicines induced late apoptosis which is more pronounced in the *Mshikazi* extract. The membrane blebbing that has been observed during the culture of cancer cells which follows the induction of apoptosis can be a result of a lack of neighbouring phagocytes or late stage of cell death 38. From our results, we found that the induction of apoptotic cells was more in Jurkat lymphocytes cells than THP-1 monocytes cells.

At the late stages of apoptosis, T lymphocytes can strip down into smaller membrane-bound extracellular vesicles called apoptotic bodies 39. The strip down of these T lymphocytes into apoptotic bodies is a very organised process induced by the simultaneous formation of membrane blebbing and apoptopodia whereas THP-1 monocyte cells show membrane blebbing morphology at about 90 minutes after the induction of apoptosis unlike the far-reaching effect in morphological change with lymphocytes cells undergoing membrane blebbing 39.

Cell death occurs in two forms, necrosis and apoptosis. Necrosis is an injury form of cell death which occurs as a result of environmental distress and causes cellular inflammation while apoptosis is programmed cell death^40^. Apoptosis is an essential measure for a potential anticancer agent. To confirm what type of cell death was involved with the THP-1 and Jurkat cells, flow cytometry analysis was evaluated using propidium iodide (PI) and JC-10. During apoptosis, the cell membrane of the cells becomes irregular and loses its integrity which results in the translocation of phosphatidylserine (PS) to the outer membrane^41^. The mitochondrial membrane potential is one of the main indicators of mitochondrial membrane integrity of a healthy cell; a decrease/loss of this potential membrane integrity leads to early apoptosis^42^. These two processes were achieved by using PI and JC-10 to stain the THP-1 and Jurkat cells in this study. The PI stain was able to identify both the early and late apoptotic cells and JC-10 was able to identify the loss of membrane potential which is an event of early apoptotic cells. The results obtained show that EMB, *Mshikazi* and *Delosma H* extracts were able to induce apoptosis at both early and late apoptosis with minimal necrosis.

From all indications, cell viability assays revealed EMB, *Mshikazi* and *Delosma H* to inhibit the growth of THP-1 and Jurkat cells; the caspase activity and flow cytometry an indication that these three extracts induced cell death (apoptosis) within 6-24 hours of incubation. A study conducted by Nordin et al. (2017) reported that the methanol extract from the leaves of Ardisia crispa induced apoptosis within 6-24 hours compared to chemotherapy drugs after staining with PI/Annexin V. This finding agreed with our study where the three extracts, especially Mshikazi, induced fast apoptosis within the incubation period of 6-24 hours. In another report, pinostrobin (PN) which is a naturally occurring bioflavonoid from medicinal herbs, induced apoptosis in HeLa cells by showing a significant increase in the JC-1 dye and a reduction in mitochondria membrane potential 42. EMB, *Mshikazi* and *Delosma H* extracts also induced cell death after staining with JC-10 dye.

The LC-MS results of EMB, *Mshikazi* and *Delosma H* extracts show different chemical compounds to be present in the three extracts. Some of the molecules are common to all three extracts while some are unique to an extract. The molecule syrosingopine was found in both EMB and *Delosma H* extracts. A study showed syrosingopine to synergistically interact with Metformin, a diabetic drug used to fight against a wide range of cancer cells including leukaemia cells in vitro 43. The compounds gamabufotalin and bufotalin are bufadienolides that were found in *Mshikazi* and *Delosma H* extracts and are known to inhibit cancer cells in vitro and in vivo, according to a report that shows their ability to inhibit the cell viability of esophageal squamous cell carcinoma with IC50 values of 0.8-3.6µM, and induced apoptosis through DNA fragmentation and nuclear condensation 44. Gamabufotalin was said to induce apoptosis through the activation of cytochrome c and the caspase-dependent apoptotic pathway in lung cancer 45. Another compound of interest is the presence of digitoxin in the EMB and Mshikazi extract. A review concluded that digitoxin and its analogues are promising anticancer agents against many types of cancers including leukaemia 46. Based on these findings, it was found that all three extracts of traditional herbal medicines possess compounds which have anticancer effects.

## Conclusion

The anticancer activity of three extracts of EMB, *Mshikazi* and *Delosma H* were examined and were found to inhibit the growth of leukaemia cancer cells in vitro and also induced apoptosis through the loss of membrane and mitochondrion integrity. LC-MS analysis showed the presence of many potent anticancer compounds such as gamabufotalin, bufotalin, syrosingopine and digitoxin which have previously been reported to possess anti-cancer effects. Out of the three extracts of traditional herbal medicines in this study, *Mshikazi* was found to possess more potent anti-leukaemia activity than EMB and *Delosma H*.

## Data Availability

All data generated or analysed during this study are included in this published article and its supplementary information files.

## Abbreviations

ATP: Adenosine triphosphate
EMB: Emelia M
LC-MS: Liquid chromatography-mass spectrophotometry
PBMCs: Peripheral blood molecular cells
S: Supplementary
THMs: Traditional herbal medicines
THPs: Traditional health practitioners

## Declarations

Biomedical Research Ethics Committee Reference No: BE367/17
